# Supplementary material for: Regressing away common neural choice signals does not make them artifacts: Comment on Frömer et al. 2024
Source: Imaging Neurosci (Camb). 2025 Jun 24;3:IMAG.a.60. doi: 10.1162/IMAG.a.60 (PMC12319789; doi:10.1162/IMAG.a.60)
Supplement: Supplementary Material [file imag.a.60_supp.pdf]

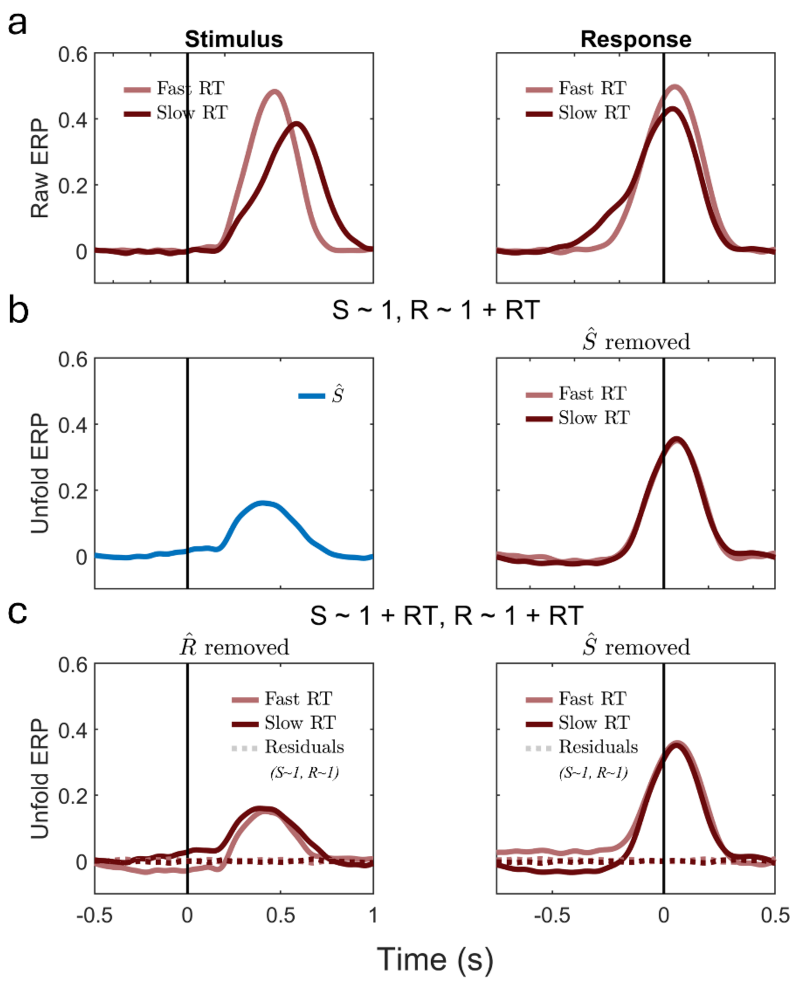

Supplementary Figure 1. *Replication of all analyses reported in Figure 1 but this time applied to a noisy diffusion process simulated from the same code used to generate Frömer et al's Supplementary Figure 5.* ***a)*** *Trial-averaged timecourses aligned to stimulus and response exhibit RT-dependent effects on both the buildup rate and amplitude reached at response****.*** ***b)*** *Unfold assigns a substantial portion of the EA signal to the S component (left panel). Removal of that S-component dramatically reduces the real RT effects on both slope and amplitude in the response-aligned trace, as observed in the empirical data analyses of Frömer et al (see Figure 6 of Frömer et al 2024).* ***c)*** *Spurious reduction of effects was also observed when allowing both the S and R components to vary with RT. Removing the estimated S and R components from an RT-agnostic regression produces virtually flat residuals, as observed in the empirical data analyses of Frömer et al. (Supplementary Figure 7 of Frömer et al. 2024). Whether or not residual activity consistent with an evidence accumulation process can be found after applying unfold may be dependent on specific data features (e.g. RT distribution, stimulus/motor variability, etc) and Unfold settings. However the key message of this simulation is that Unfold can result in flat residuals even for a ground truth EA signal, and therefore Frömer et al's results are not informative regarding the presence or absence of EA signals in their empirical data.*
